# So Small, So Loud: Extremely High Sound Pressure Level from a Pygmy Aquatic Insect (Corixidae, Micronectinae)

**DOI:** 10.1371/journal.pone.0021089

**Published:** 2011-06-15

**Authors:** Jérôme Sueur, David Mackie, James F. C. Windmill

**Affiliations:** 1 Muséum national d'Histoire naturelle, Département Systématique et Evolution, UMR 7205 CNRS Origine Structure et Evolution de la Biodiversité, Paris, France; 2 Department of Electronic and Electrical Engineering, Centre for Ultrasonic Engineering, University of Strathclyde, Glasgow, United Kingdom; University of Maryland, United States of America

## Abstract

To communicate at long range, animals have to produce intense but intelligible signals. This task might be difficult to achieve due to mechanical constraints, in particular relating to body size. Whilst the acoustic behaviour of large marine and terrestrial animals has been thoroughly studied, very little is known about the sound produced by small arthropods living in freshwater habitats. Here we analyse for the first time the calling song produced by the male of a small insect, the water boatman *Micronecta scholtzi*. The song is made of three distinct parts differing in their temporal and amplitude parameters, but not in their frequency content. Sound is produced at 78.9 (63.6–82.2) SPL rms *re* 2.10^−5^ Pa with a peak at 99.2 (85.7–104.6) SPL *re* 2.10^−5^ Pa estimated at a distance of one metre. This energy output is significant considering the small size of the insect. When scaled to body length and compared to 227 other acoustic species, the acoustic energy produced by *M. scholtzi* appears as an extreme value, outperforming marine and terrestrial mammal vocalisations. Such an extreme display may be interpreted as an exaggerated secondary sexual trait resulting from a runaway sexual selection without predation pressure.

## Introduction

Animal communication is driven by competition between individuals and species [1]–[3]. The signal produced by an emitter should reach as many receivers as possible, whilst transmitting as much information as possible. To increase the range of their broadcast, animals can optimize the ratio of their signal to the background noise. One of the simplest strategies to achieve this is to produce a signal with a high amplitude that can override congener or other species songs, travelling the greatest distance across the habitat [4]. When considering acoustic communication, the production of a loud, and intelligible, signal is not an easy task even for human-built sound systems [5]. The system can be over-driven, distorting time and frequency parameters, and consequently impairing information transfer. In addition, animals are severely constrained by their morphological characteristics. Body size is one of the main mechanical constraints as a small sound source cannot produce a high level sound output [6], [7]. This phenomenon explains why large mammals, such as whales or elephants, are known to be the loudest animals [8], [9]. However, when these animals are scaled to their body size they may not produce the most efficient acoustic signals in terms of energy.

Acoustic communication is intensively studied in terrestrial and marine animals, but is neglected in freshwater species even when low visibility should favour acoustics as a way to exchange information. There are potentially an important number of aquatic insects that can sing underwater, but very few descriptions of their behaviour have been reported [10]–[16]. Water-boatman species belonging to the genus *Micronecta* (Corixidae, Micronectinae) are known to use sound for pair formation [14], [15]. Only males produce species-specific sounds that attract females for mating [16]–[18]. Males can synchronize their calls generating a chorus [19]. This suggests a possible second role of male-male competition as observed in several other insects using sound to court females [2]. Here we report for the first time the acoustic behaviour of *Micronecta scholtzi* (Fieber, 1860), a common aquatic bug that produces an extremely loud courtship song. This insect is a few millimetres in length yet can produce sound audible from the riverside. This suggests the emission of intense signals departing from the body size to amplitude rule.

## Materials and Methods

Specimens of *M. scholtzi* were collected in a river in Paris (France, 48°49.42′N–02°25.93′E) and in a pond in Morsang-sur-Orge (France, 48°40.03′N–02°20.59′E) from August to September 2009 and 2010. According to the national guidelines, no permission was required from authorities to collect specimens. Specimens were maintained in plastic water tanks (22*11*17 cm). Sex determination was not possible without manipulating individuals. As *M. scholtzi* is active only in groups, samples of five unsexed individuals were transferred to a fish net breeder (16.5*12.2*13 cm) which was positioned at the centre of a large plastic water tank (46*30*17 cm) with a water depth of 8 cm. The bottom of the tank was covered with gravel without any plants. This recording area provided a short distance between the insects and the hydrophone, and a relatively large distance between the hydrophone and the tank walls. This minimized sound wave reflections that could have impaired recording quality. A Reson TC4033 passive hydrophone was placed at the bottom of the net breeder, in the centre. The hydrophone was connected to an Avisoft charge amplifier with an input capacitance of 1 nF and a 250 Hz high-pass input filter. Recordings were taken with a Marantz PMD 671 digital recorder at 48 kHz sampling frequency and 16 bit level digitization. All recordings were made at a water temperature of 23–24°C controlled with a Tetratex HT50 heater.

As *Micronecta* females do not produce sound [15], all sound recorded was considered as being produced by males. The calling songs of 13 males were recorded and 60 seconds of signal without background were selected for each male. Even if *M. scholtzi* call in chorus there is no fine synchronization of their signals. It was then possible to select a signal produced by a single animal excluding the risk of analysing several males together. Calling song parameters were analysed using *Avisoft SAS Lab Pro* [20] and *seewave* [21]. Temporal parameters were measured on the amplitude envelope. Frequency parameters were measured on the mean spectrum of a short-term Fourier transform with a frequency resolution of 43 Hz.

In order to produce an accurate measure of the sound-pressure-level (SPL), the recording equipment (hydrophone+charge amplifier+digital recorder) was calibrated in reference to a sound source emitting a signal at a known SPL. This was achieved by using one passive hydrophone (Reson TC4013) as an emitter and a second passive hydrophone as a receiver (Reson TC4033). This receiver hydrophone was connected to an Avisoft charge amplifier with an input capacitance set to 1 nF and a 250 Hz high-pass input filter, which in turn was connected to a Marantz PMD 671 digital recorder. The recording chain was therefore exactly the same as the one used to record the animals. The emitter output was a 10 kHz sine wave that was repeated for different acoustic amplitudes and for different Marantz PMD 671 manual recording input levels. Peak and root-mean-square (rms) of the digital values of the amplitude envelope were then calculated for each *M. scholtzi* recording selection. Average values were computed on this raw data before being converted to dB SPL in reference to 2.10^−5^ Pa to allow comparison with terrestrial animals (see below). As the distance between the animal and the hydrophone was not known, three estimations were assessed assuming the distance was minimal (0.05 cm), median (6.5 cm) or maximal (13 cm). Male body length was measured after recordings using the graticule of a binocular microscope Leica M205C with a precision of ±0.05 mm.

SPL values of *M. scholtzi* were compared with the values reported for 227 other species (2 reptiles, 3 fishes, 24 mammals, 29 birds, 46 amphibians and 123 arthropods) collected from the literature (Table S1). This includes 17 species (7.5% of 227) for which SPL values were estimated underwater. Two of this latter group were arthropods, namely the Crustaceans *Panulirus interruptus* and *Synalpheus parneomeris* (0.9% of 227). Only communication signals were considered, echolocation or debilitating sound was excluded. Different SPL values could be found for a single species. These values may come from different references or from variability across populations, sexes and signal types within a repertoire. The highest dB SPL value was selected in all cases. Peak measurements were converted into rms measurements by dividing them by √2 [22]. The SPL values found in the literature are all given in dB. However, they refer to measurements done at a different distance *d* and/or in reference to a different reference pressure *P_0_*. To allow comparison across taxa, all data were first converted to sound pressure (Pa). Sound pressure data were then converted back to dB SPL with a reference pressure *P_0_* = 2.10^−5^ Pa. Data were eventually converted to SPL data at a distance of 1 m by applying the attenuation inverse square law following the equation [23]:
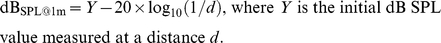



Body length estimation was also documented for all species. As dB is a logarithmic scale, and as sound pressure scales with body mass rather than body length [5], the link between dB SPL and animal size was estimated between dB and the logarithm of body length cubed (*i.e.* 3×log_10_(body length)). This was achieved for two sub-samples corresponding to the main characteristics of *M. scholtzi* acoustic communication system. The first sub-sample included all stridulating animals (57 arthropods and one fish). The second sub-sample included all underwater animals (three arthropods, three fish and 11 mammals). Because of the presence of outliers in the sample, both ordinary least squared (OLS) and robust regressions were computed [24], [25]. All statistics were run using *R* with the additional package *robust* [26].

## Results

**Figure 1:**
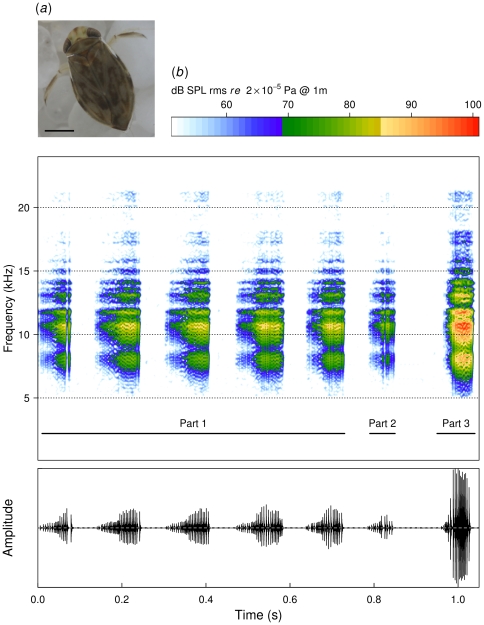
Habitus and calling song of *M. scholtzi*. (*a*) dorsal view of an adult (scale bar = 0.5 mm), (*b*) calling song consisting of three main parts differing in their temporal and amplitude parameters (oscillogram), but having a similar frequency structure (spectrogram and amplitude scale with an estimated maximum value of 101 dB SPL rms *re* 2.10^−5^ Pa at 1 meter).

The size of the *M. scholtzi* male was 2.3±0.1 mm (mean ± s.d., *n* = 21) (Fig. 1). The song consisted of a typical sequence repeated at a rate of 0.746±0.129 Hz (*n* = 582). Each sequence was composed of three parts differing in their temporal and amplitude parameters (Fig. 1). The first part was a repetition of 5.1±1.4 (*n* = 582) quiet echemes that lasted 84±19 ms (*n* = 2994) and were followed by a silence of 192± c cvv48 ms (*n* = 2994). The second part was a succession of 1.6±0.7 (*n* = 582) short and quiet echemes that lasted 16±3 ms (*n* = 820) followed by a silence of 101±21 ms (*n* = 820). The third part was a single loud echeme of a duration of 60±8 ms (*n* = 582). The frequency spectrum extended from 5 to 22 kHz with 50% of the signal energy between 9 to 11 kHz with a dominant frequency at around 10 kHz (1^st^ part: 10.063±1.122 kHz (*n* = 2994); 2^nd^ part: 10.348±0.872 kHz (*n* = 820); 3^rd^ part: 10.109±0.886 kHz (*n* = 582)). There was no frequency modulation along the signal, the frequency content of the different parts being similar.

The minimal, median and maximal amplitude level of the song were respectively estimated to be 36.7 (21.5–39.9) (mean (min – max)), 78.9 (63.6–82.2) and 85.0 (69.6–88.2) dB SPL rms *re* 2.10^−5^ Pa at 1 meter. Peak values were estimated to be 57.1 (43.6–88.2), 99.2 (85.7–104.6) and 105.2 (91.7–110.6) dB SPL rms *re* 2.10^−5^ Pa at 1 metre.

**Figure 2:**
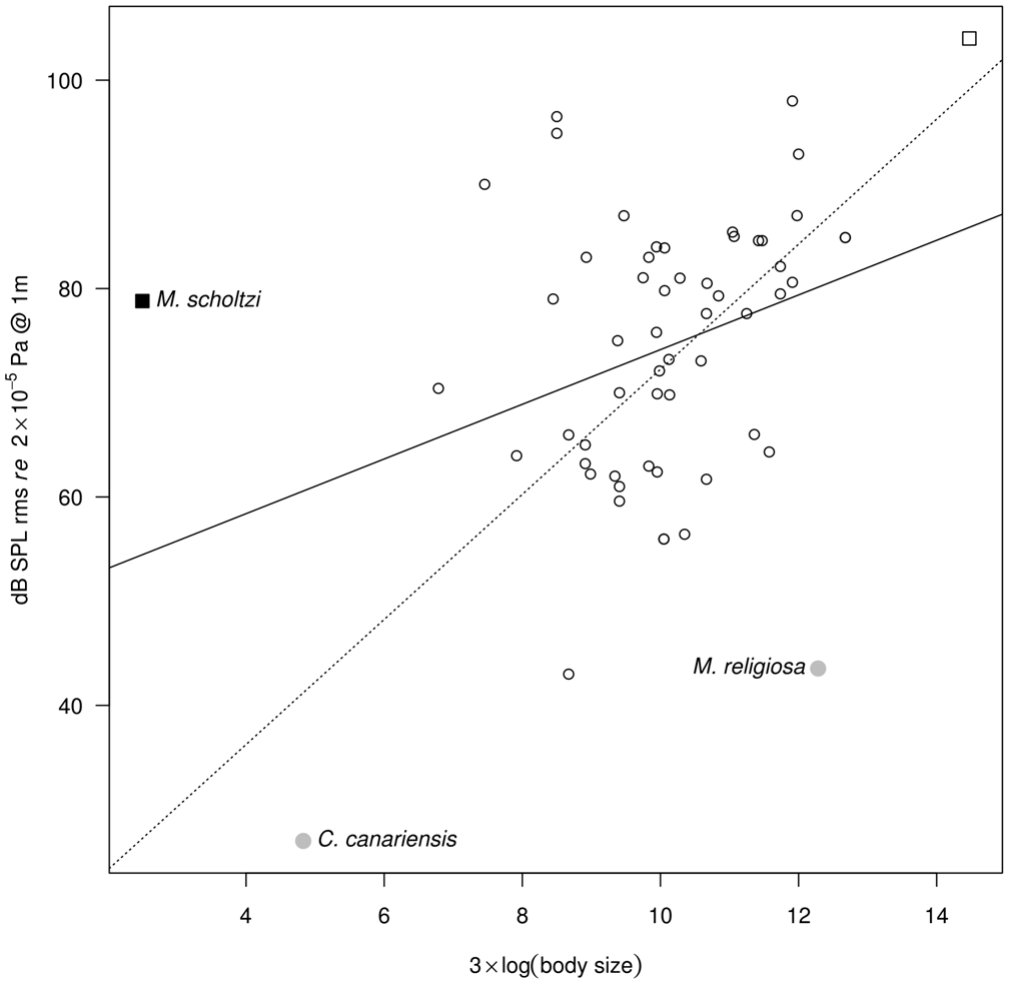
Regression between body size and SPL for stridulating animals. Terrestrial species are indicated with a circle and underwater species with a square. The species labelled with a plain symbol are identified as outliers following Cook's distance and leverage (electronic supplementary material, figures S1, S2). Regression lines: ordinary least squared regression (plain) and robust regression (dashed). Sample size: 57 arthropods and one fish.

The average of the ratio dB/(3×log_10_(body length)) for all animals documented was 6.9±3.0 (*n* = 228). A maximum value of 31.5 was estimated for *M. scholtzi*. Within the group of 58 stridulating animals, the OLS regression against dB and 3×log_10_(body length) indicated the following three species as outliers: (i) *M. scholtzi*, (ii) the miniature cricket *Cycloptiloides canariensis*, and (iii) the praying mantis *Mantis religiosa* (Fig. 2, F_1,56_ = 7.44, R^2^ = 0.10, *p* = 0.009). *M. scholtzi* was isolated due to its high SPL and small size, while *C. canariensis* was isolated by its small size and low SPL, and *M. religiosa* by a particularly low SPL compared to its large size. Cook's distance associated with species leverage on the OLS model clearly identified *M. scholtzi* as the most extreme outlier (Figs. S1, S2). A robust regression, which is less sensitive to outliers, returns a regression line with a higher regression coefficient (Fig. 2, F_1,56_ = 6.23, R^2^ = 0.33, *p* = 0.011).

**Figure 3:**
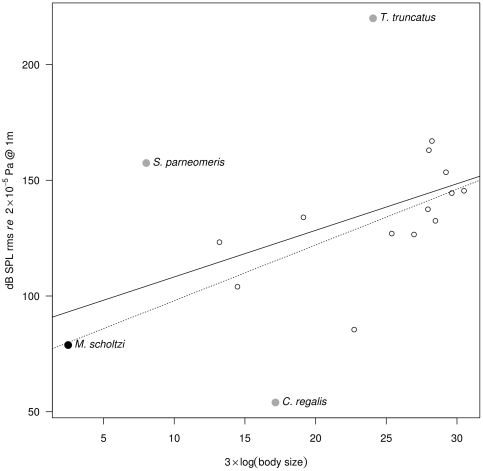
Regression between body size and SPL for underwater animals. The species labelled with a plain symbol are identified as outliers following Cook's distance and leverage (electronic supplementary material, figures S3, S4). Regression lines: ordinary least squared regression (plain) and robust regression (dashed). Sample size: three arthropods, three fish, 11 mammals.

Within the group of animals using sound underwater, the OLS regression had a p-value just above a 5% α risk (Fig. 3, F_1,15_ = 3.60, R^2^ = 0.14, *p* = 0.077). The OLS regression indicated the following four species as outliers: (i) the snapping shrimp *Synalpheus parneomeris*, (ii) the weakfish *Cynoscion regalis*, (iii) the common bottlenose dolphin *Tursiops truncatus*, and (iv) *M. scholtzi*. *S. parneomeris* was isolated by its small size and high SPL, *C. regalis* by its medium size and low SPL, *T. truncatus* by its high SPL, and *M. scholtzi* by its very small size (Figs. S3, S4). A robust regression was significant and returned a regression line with a higher regression coefficient (Fig. 3, F_1,15_ = 5.52, R^2^ = 0.33, *p* = 0.017).

## Discussion

The water boatman *M. scholtzi* produces a complex calling song comprising three distinct parts with deep amplitude modulations, but no frequency modulation. However, the most striking feature of the song is its intensity. The song can be heard by a human ear from the side of a pond or river, propagating across the water-air interface. Estimating the sound intensity at a distance of one metre reveals a value of ∼79 dB SPL rms. When considering peak values, *i.e.* the loudest part of signal, the intensity can reach 100 dB SPL. Whilst these values are far below those estimated for large mammals such as dolphins, whales, elephants, hippos, or bison, when scaled to body size, *M. scholtzi* has the highest ratio dB/body size. Even if such comparison might need to be adjusted with corrections taking into account different recording methods and conditions, *M. scholtzi* is clearly an extreme outlier with a dB/body size ratio of 31.5 while the mean is at 6.9 and the second highest value is estimated at 19.63 for the snapping shrimp *S. parneomeris*. This water bug might be the exception that proves the rule that stipulates that the size and the intensity of a source are positively related. This departure from the rule is apparent within the group of stridulating animals. In this sub-sample, *M. scholtzi* is identified as an extreme outlier. No other recorded animals rival *M. scholtzi*. Two other arthropods were also identified as outliers; the Australian miniature cricket *C. canariensis* [27] and the Praying Mantis *M. religiosa* [28]. In both cases these are outliers for different reasons, as the Praying Mantis emits a much quieter song (∼43 dB) than suggested by its size (∼60 mm), and the miniature cricket is particularly small (∼3 mm) and quiet (∼30 dB). When considering aquatic animals, whatever the mode of sound production they use (*i.e.* drumming, friction, stridulation or vocalisation), *M. scholtzi* appears as an outlier mainly due to its very small size compared to fish, mammals or even crustacean species communicating underwater. Producing loud sound underwater is easier than in the air due to impedance-matching between the source, here the body part of the animal that generates vibrations, and the transmission media (water) [23]. This might explain why *M. scholtzi* appears as the most extreme outlier when compared to stridulating species that are terrestrial (except one fish), and is identified as only the fourth outlier when considering underwater species. Oxygen uptake of *Micronecta* has not been studied in detail but air is stored around their body by hydrofuge hairs. The ventral side is indeed covered with an air layer [29]. This suggests that the stridulating mechanism might be in contact with air but not water. This could induce a complex micro acoustic environment with reflections and refraction due to impedance differences between air and water.

The mechanism behind the intense sound production of *M. scholtzi* is not clearly identified. The sound is produced by rubbing a *pars stridens* on the right paramere (genitalia appendage) against a ridge on the left lobe of the eighth abdominal segment [15]. This sound emission system does not measure more than 50 µm in length, and there are no obvious body or external resonating systems that could amplify the sound, as observed in insects, amphibians, mammals and birds [30]–[35]. The high sound output (∼124 dB) observed in *Panulirus* spiny lobsters has been explained by the use of stick-slip friction instead of a classical stridulation [36], [37]. This mechanism might occur in *M. scholtzi*, but to observe the micro-mechanics of such a small system remains a significant challenge.

Could we try to interpret why *M. scholtzi*, and presumably other *Micronecta* species [17], produce such loud sounds? An increase of signal amplitude in reaction to a rise of the background noise, known as the Lombard effect, has been documented for various birds and mammals, including man [4]. However, this amplitude rise is only observed over the short-term. Here the high amplitude level is a long-term process that might result from intra-specific competition. *Micronecta* male stridulation has been proven to be a sexual signal addressed to the female, which can use the signal to select a conspecific male (species identification) [17] and to select a male among other conspecific males (sexual selection) [15]. The extreme SPL level of this signal could be compared to the extremely high complexity of some bird songs, particularly long mammal antlers, complex insect horns, or the brightly coloured integumentary system found in almost all animal groups. All of these exaggerated secondary sexual ornaments are thought to be a by-product of a runaway or Fisherian sexual selection [38], [39], especially in the case of insect acoustic signals [40], [41]. A signal produced at high amplitude can potentially override the signals emitted by competitors during chorusing bouts and hence facilitate male localisation by the choosing female [1]–[3], [42]. Acoustic competition can then lead to loud signal levels. However, such a runaway process can be counterbalanced by natural selection if the extreme signal tends to have adverse effects. The extreme signal might be too costly in terms of energy or too risky in terms of predation. An obvious acoustic display could attract predators that localise their prey through audition [43], [44]. Predators and parasitoids can strongly constrain song evolution and can even lead to a disappearance of the acoustic sexual signal [45]. Nothing is known about predation on *M. scholtzi*, but the extreme SPL value suggests the absence of such an evolutionary limiting factor. Male of *M. scholtzi* may have no auditory predator, or escape such a predator more efficiently than other acoustic species. The hypothesis of a runway selection being at the origin of *M. scholtzi* loudness still needs to be tested with observations on competition behaviour between males and with an estimation of the predator guild associated with males. Eventually, playback experiments based on the broadcast of pairs of similar signals with similar time and frequency pattern, but different SPL values, could test female preference for loud over soft calls.

## Supporting Information

Figure S1 **Cook's distance of each of the 58 stridulating animals (57 arthropods and one fish) included in an OLS model.** Three species were identified by the model: the praying mantis *Mantis religiosa*, the miniature cricket *Cycloptiloides canariensis* and the water-boatman *Micronecta scholtzi*.
(TIFF)

Figure S2 **Scatterplot of leverage and standardized residuals of the model.** As in Fig. S1, the praying mantis *Mantis religiosa*, the miniature cricket *Cycloptiloides canariensis* and the water-boatman *Micronecta scholtzi*. *M. scholtzi* has the highest leverage.
(TIFF)

Figure S3 **Cook's distance of each of the 17 animals calling underwater (freshwater or marine habitats) included in an OLS model.** Four species were identified by the model: the snapping shrimp *Synalpheus parneomeris*, the weakfish *Cynoscion regalis*, the common bottlenose dolphin *Tursiops truncatus* and the water-boatman *Micronecta scholtzi*.
(TIFF)

Figure S4 **Scatterplot of leverage and standardized residuals of the model.** As in Fig. S3, the following four species are identified as outliers: the snapping shrimp *Synalpheus parneomeris*, the weakfish *Cynoscion regalis*, the common bottlenose dolphin *Tursiops truncatus* and the water-boatman *Micronecta scholtzi*.
(TIFF)

Table S1 **Species list and references used to assess sound pressure level (dB L) and body size relationship.** Underwater recordings are denoted with an asterisk (*) before species name.
(PDF)
