# Supplementary figures and images for: So Small, So Loud: Extremely High Sound Pressure Level from a Pygmy Aquatic Insect (Corixidae, Micronectinae)
Source: PLoS One. 2011 Jun 15;6(6):e21089. doi: 10.1371/journal.pone.0021089 (PMC3115974; doi:10.1371/journal.pone.0021089)

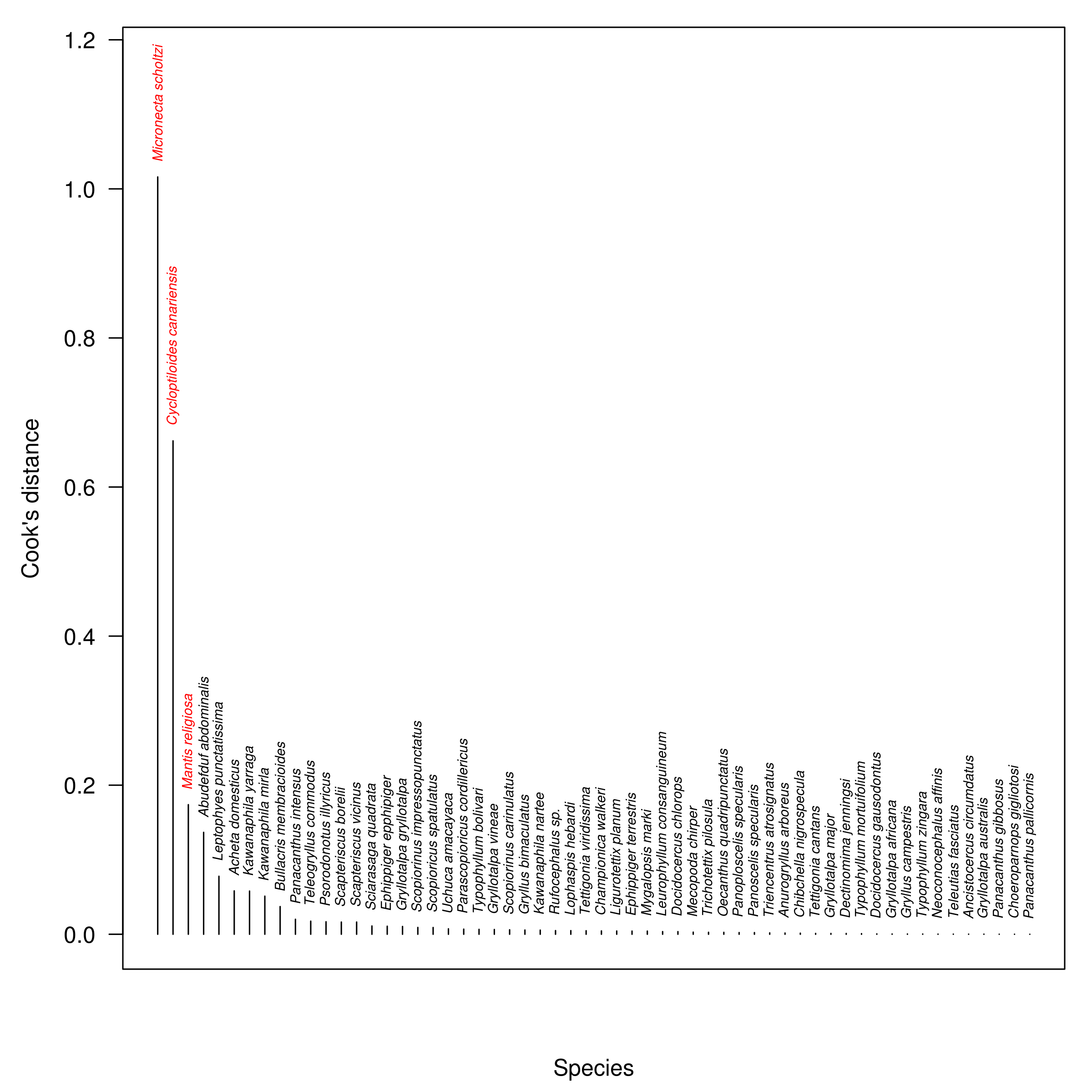

Supplement: Figure S1 — Cook's distance of each of the 58 stridulating animals (57 arthropods and one fish) included in an OLS model. Three species were identified by the model: the praying mantis Mantis religiosa, the miniature cricket Cycloptiloides canariensis and the water-boatman Micronecta scholtzi. (TIFF) [file pone.0021089.s001.tif]

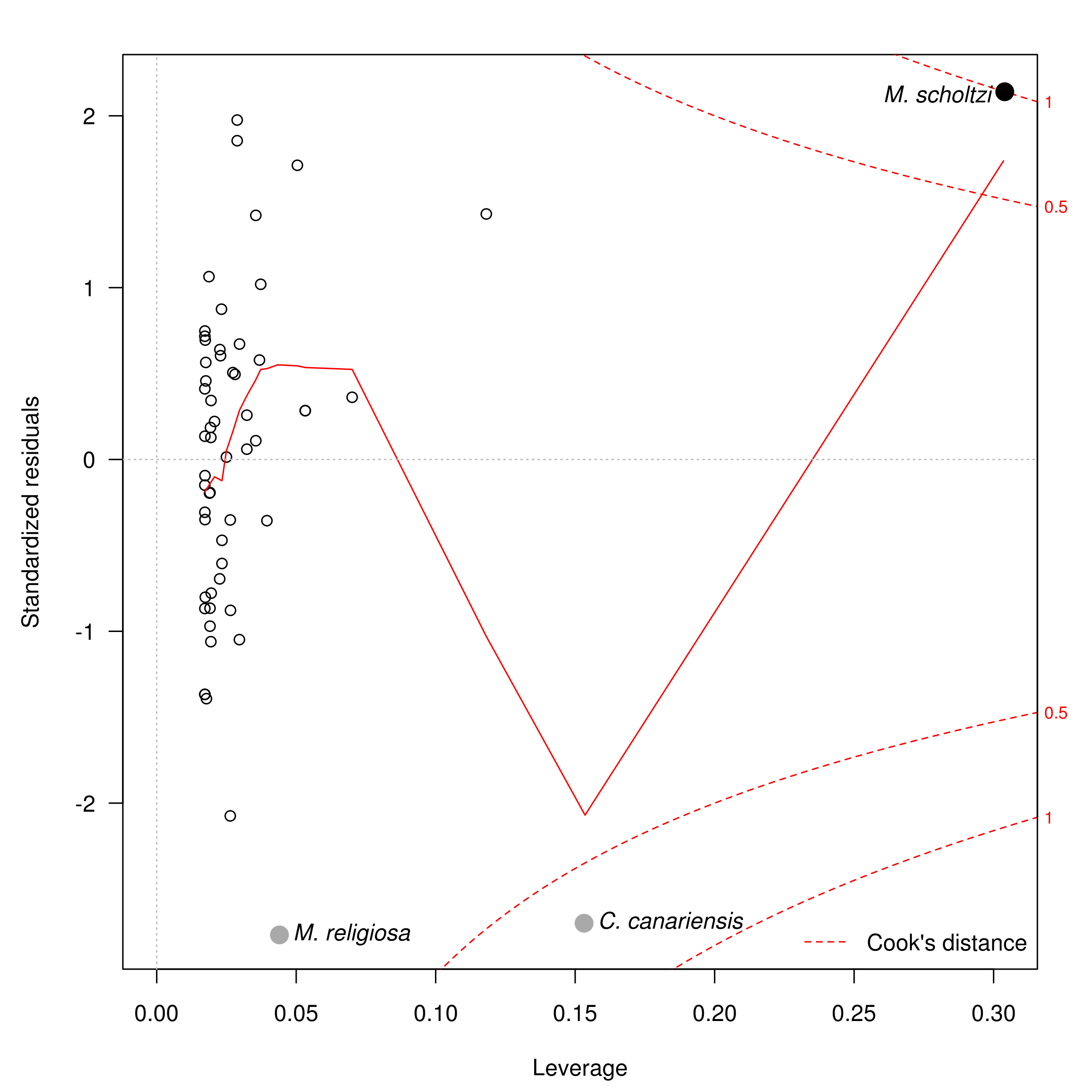

Supplement: Figure S2 — Scatterplot of leverage and standardized residuals of the model. As in Fig. S1, the praying mantis Mantis religiosa, the miniature cricket Cycloptiloides canariensis and the water-boatman Micronecta scholtzi. M. scholtzi has the highest leverage. (TIFF) [file pone.0021089.s002.tif]

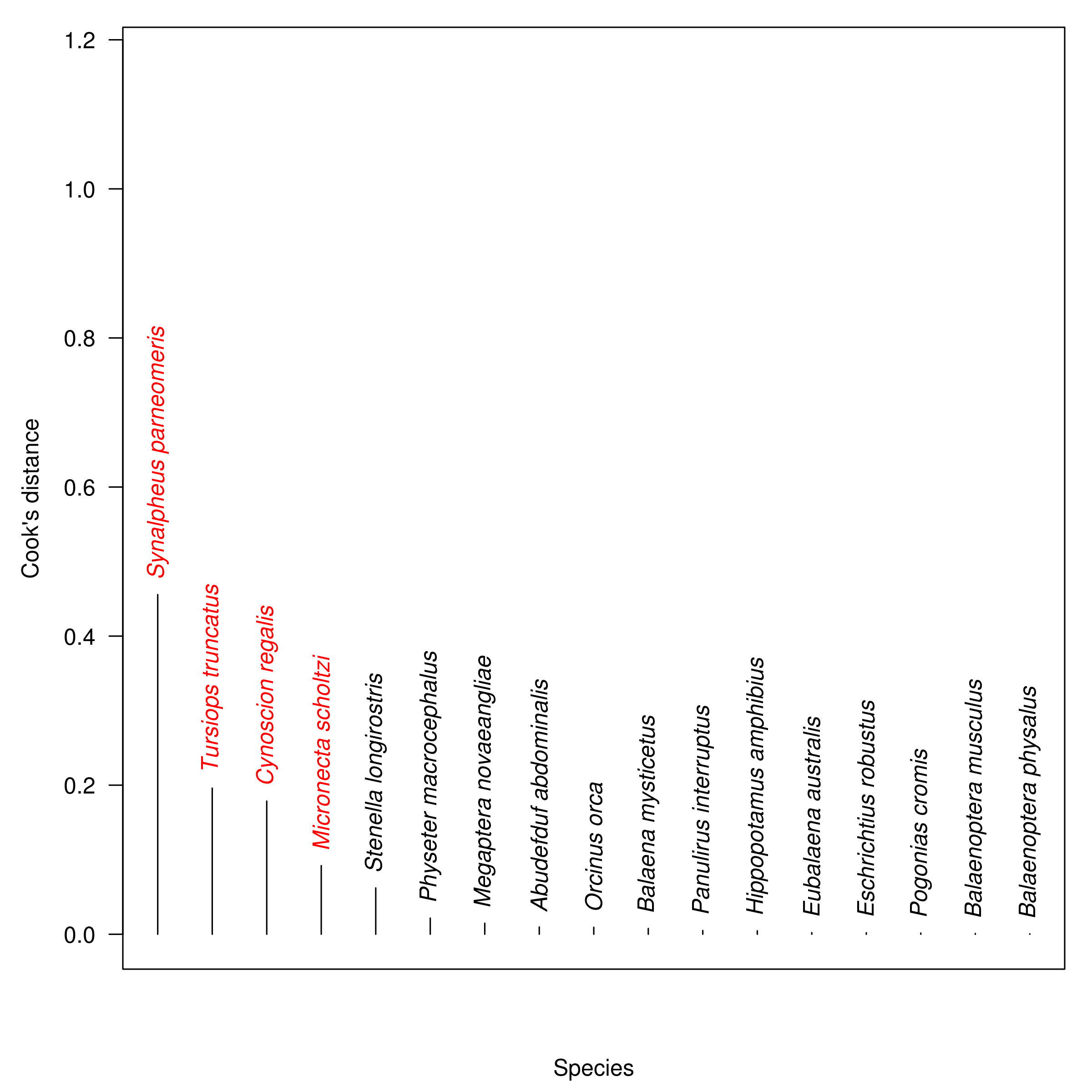

Supplement: Figure S3 — Cook's distance of each of the 17 animals calling underwater (freshwater or marine habitats) included in an OLS model. Four species were identified by the model: the snapping shrimp Synalpheus parneomeris, the weakfish Cynoscion regalis, the common bottlenose dolphin Tursiops truncatus and the water-boatman Micronecta scholtzi. (TIFF) [file pone.0021089.s003.tif]

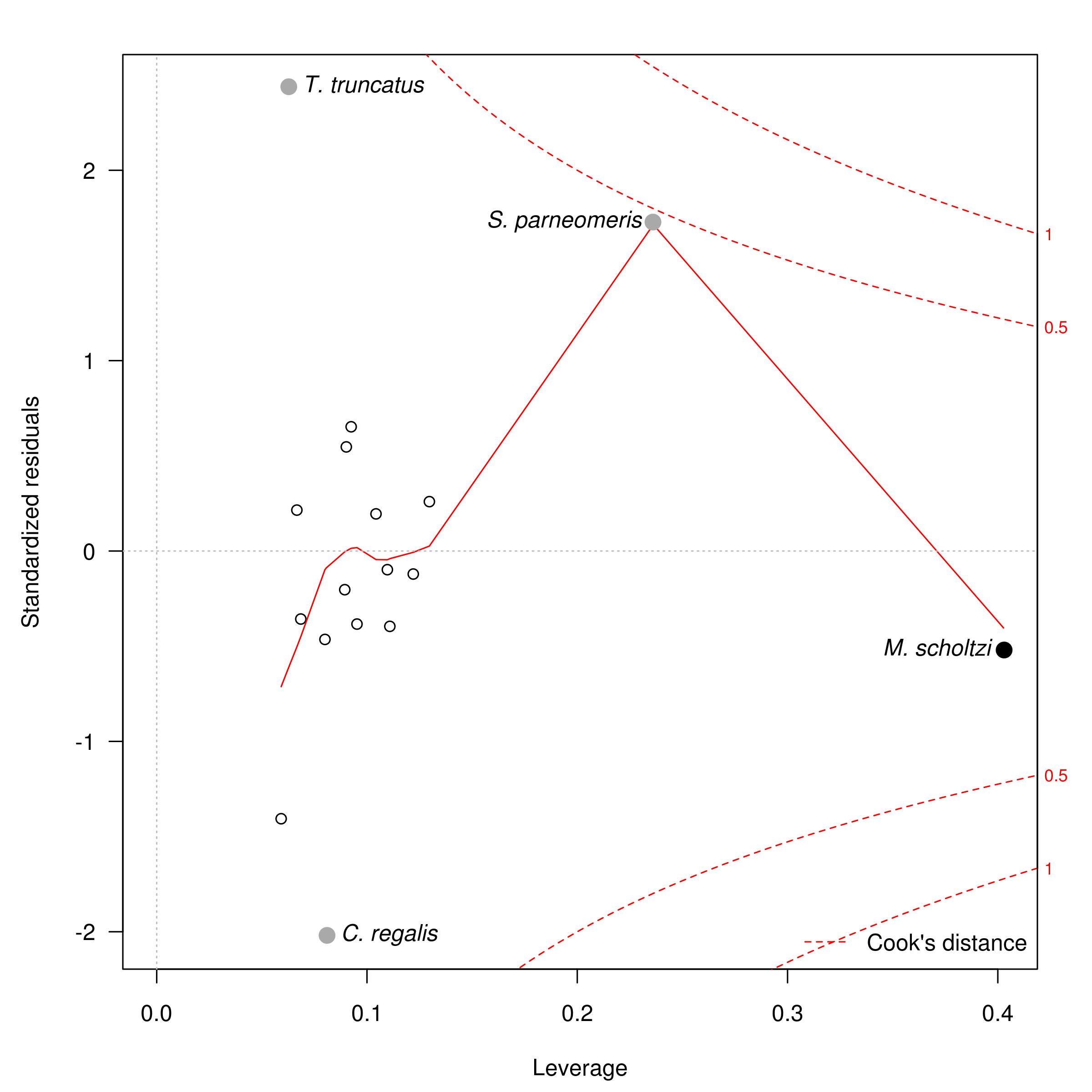

Supplement: Figure S4 — Scatterplot of leverage and standardized residuals of the model. As in Fig. S3, the following four species are identified as outliers: the snapping shrimp Synalpheus parneomeris, the weakfish Cynoscion regalis, the common bottlenose dolphin Tursiops truncatus and the water-boatman Micronecta scholtzi. (TIFF) [file pone.0021089.s004.tif]
