# Supplementary material for: So Small, So Loud: Extremely High Sound Pressure Level from a Pygmy Aquatic Insect (Corixidae, Micronectinae)
Source: PLoS One. 2011 Jun 15;6(6):e21089. doi: 10.1371/journal.pone.0021089 (PMC3115974; doi:10.1371/journal.pone.0021089)
Supplement: Table S1 — Species list and references used to assess sound pressure level (dB L) and body size relationship. Underwater recordings are denoted with an asterisk (*) before species name. (PDF) [file pone.0021089.s005.pdf]

Table S1 - Species list and references used to assess sound pressure level (dB SPL) and body size relationship. Underwater recordings are denoted with an asterisk (*) before species name.

Amphibian

Acris crepitans	Gerhardt (1975)	Gerhardt (1975)
Afrixalus brachycnemis	Passmore (1981)	Passmore (1981)
Afrixalus fornasinii	Passmore (1981)	Passmore (1981)
Batrachyla antartandica	Penna & Solis (2005)	Penna & Solis (2005)
Batrachyla leptopus	Penna & Solis (2005	Penna & Solis (2005)
Buffo gutturalis	Passmore (1981)	Passmore (1981)
Bufo americana	Gerhardt (1975)	Conant & Collins (1991)
Bufo garmani	Passmore (1981)	Passmore (1981)
Bufo quercicus	Gerhardt (1975)	Conant & Collins (1991)
Bufo terrestris	Gerhardt (1975)	Conant & Collins (1991)
Cacosternum bettgeri	Passmore (1981)	Passmore (1981)
Eleutherodactylus coqui	Narins & Hurley (1982)	Narins & Hurley (1982)
Engystomops pustulosus	Ryan (1985)	Wilczynski et al. (2001)
Eupsophus emiliopugini	Penna & Solis (2005	Penna & Solis (2005)
Gastrophryne carolinensis	Gerhardt (1975)	Conant & Collins (1991)
Hyla arborea	Marquez et al. (2005)	Marquez et al. (2005)
Hyla avivoca	Gerhardt (1975)	Conant & Collins (1991)
Hyla cinerea	Gerhardt (1975)	Conant & Collins (1991)
Hyla crysoscelis	Gerhardt (1975)	Conant & Collins (1991)
Hyla femoralis	Gerhardt (1975)	Conant & Collins (1991)
Hyla gratiosa	Gerhardt (1975)	Conant & Collins (1991)
Hyla meridionalis	Marquez et al. (2005)	Marquez et al. (2005)
Hyla squirella	Gerhardt (1975)	Conant & Collins (1991)
Hyla versicolor	Gerhardt (1975)	Conant & Collins (1991)
Hylorina sylvatica	Penna & Solis (2005)	Penna & Solis (2005)
Hyperolius argus	Passmore (1981)	Passmore (1981)
Hyperolius marmoratus	Passmore (1981)	Passmore (1981)
Hyperolius pusillus	Passmore (1981)	Passmore (1981)
Hyperolius tuberilinguis	Passmore (1981)	Passmore (1981)
Kassina maculata	Passmore (1981)	AmphibiaWeb (2010)
Limnaoedus ocularis	Gerhardt (1975)	Wright & Wright (1949)
Lithobates virgatipes	Gerhardt (1975)	Conant & Collins (1991)
Phrynobatrachus mababiensis	Passmore (1981)	Lambiris (1989)
Phrynobatrachus natalensis	Passmore (1981)	Passmore (1981)
Phrynomerus bifasciatus	Passmore (1981)	Passmore (1981)
Pleurodema thaul	Penna & Solis (2005)	Penna & Solis (2005)
Pseudacris crucifer	Gerhardt (1975)	Conant & Collins (1991)
Pseudacris streckeri	Gerhardt (1975)	Conant & Collins (1991)
Pseudacris triseriata	Gerhardt (1975)	Conant & Collins (1991)
Ptychadena anchietae	Passmore (1981)	Passmore (1981)
Ptychadena mossambica	Passmore (1981)	Passmore (1981)
Rana areolata	Gerhardt (1975)	Conant & Collins (1991)
Scaphiopus holbrooki	Gerhardt (1975)	Conant & Collins (1991)
Tomopterna cryptotis	Passmore (1981)	Passmore (1981)
Tomopterna krugerensis	Passmore (1981)	Passmore (1981)
Xenopus borealis	Yager (1992)	Yager (1992)

**Bird**		
Acrocephalus schoenobaenus	Brackenbury (1979)	Jonsson (1994)
Alauda arvensis	Briefer et al. (2008)	Jonsson (1994)
Aptenodytes patagonicus	Aubin & Jouventin (1998)	Korc & Fraser (2006)
Calonectris diomedea	Curé et al. (2009)	Jonsson (1994)
Carduelis cannabina	Brackenbury (1979)	Jonsson (1994)
Emberiza citrinella	Brackenbury (1979)	Jonsson (1994)
Emberiza shoeniclus	Brackenbury (1979)	Jonsson (1994)
Erithacus rubecula	Brackenbury (1979)	Jonsson (1994)
Eudyptes chrysolophus	Searby et al. (2004)	Reynolds (2001)
Eudyptula minor	Jouventin & Aubin (2000)	Quick (2001)
Fringilla coelebs	Brackenbury (1979)	Jonsson (1994)
Larus ridibundus	Charrier et al. (2001)	Jonsson (1994)
Locustella naevia	Brackenbury (1979)	Jonsson (1994)
Parus ater	Brackenbury (1979)	Jonsson (1994)
Phylloscopus collybita	Brackenbury (1979)	Jonsson (1994)
Phylloscopus trochilus	Brackenbury (1979)	Jonsson (1994)
Poecile atricapillus	Charrier et al. (2005)	Peterson (1980)
Puffinus tenuirostris	Jouventin & Aubin (2000)	Lindsey (1986)
Puffinus yelkouan	Curé et al. (2009)	Jonsson (1994)
Pygoscelis adeliae	Jouventin & Aubin (2002)	Combos & Francl (2008)
Pygoscelis papua	Jouventin & Aubin (2002)	Ahmed (2003)
Regulus regulus	Brackenbury (1979)	Jonsson (1994)
Strix aluco	Lengagne & Slater (2002)	Jonsson (1994)
Sylvia atricapilla	Brackenbury (1979)	Jonsson (1994)
Sylvia communis	Brackenbury (1979)	Jonsson (1994)
Sylvia curruca	Brackenbury (1979)	Jonsson (1994)
Troglodytes troglodytes	Brenowitz (1982)	Jonsson (1994)
Turdus merula	Brackenbury (1979)	Jonsson (1994)
Turdus philomelos	Brackenbury (1979)	Jonsson (1994)
**Crustacea**		
**Panulirus interruptus*	Patek et al (2009)	Patek et al (2009)
**Synalpheus parneomeris*	Au & Banks (1998)	Au & Banks (1998)
**Fish**		
**Abudefduf abdominalis*	Maruska et al (2007)	Maruska et al. (2007)
**Cynoscion regalis*	Connaughton et al (2000)	Connaughton et al. (2000)
**Pogonias cromis*	Locascio (comm. pers.)	Murphy & Taylor (1989)
**Insect**		
Acheta domesticus	Dumortier (1963)	Bellmann & Luquet (1993)
Amyna natalis	Heller & Achman (1993)	Heller & Achman (1993)
Ancistocercus circumdatus	Morris & Beier (1982)	Morris & Beier (1982)
Anurogryllus arboreus	Forrest (1991)	Walker (1973)
Ascotis selenaria	Nakano et al. (2009)	Esaki et al. (1957)
Beameria venosa	Sanborn & Phillips (1995b)	Sanborn et al. (2009)
Brevisana brevis	Villet (1987)	Villet (1987)
Bullacris membracioides	Staaden & Römer (1997)	Eades et al. (2010)
Championica walkeri	Montealegre-Z & Morris (1999)	Montealegre-Z & Morris (1999)
Chibchella nigrospecula	Montealegre-Z & Morris (1999)	Montealegre-Z & Morris (1999)
Chilo suppressalis	Nakano et al. (2009)	Esaki et al. (1957)
Choeroparnops gigliotosi	Morris et al. (1994)	Morris et al. (1994)

Crambidia pallida	Fullard & Fenton (1977)	Covell (2005)
Cyclochila australasiae	Young (1990)	Young (1990)
Cycloptiloides canariensis	Dambach & Gras (1995)	Dambach & Gras (1995)
Cycnia oregonensis	Fullard & Fenton (1977)	Covell (2005)
Cycnia tenera	Fullard (1977)	Covell (2005)
Cystosoma saundersii	MacNally & Young (1981)	Bennet-Clark & Young (1998)
Dectinomima jenningsi	Montealegre-Z & Morris (2003)	Montealegre-Z & Morris (2003)
Diceroprocta apache	Sanborn & Phillips (1995b)	Davis (1921)
Diceroprocta auriantaca	Sanborn & Phillips (2001)	Sanborn & Phillips (2001)
Diceroprocta bibbyi	Sanborn & Phillips (1995b)	Davis (1928)
Diceroprocta canescens	Sanborn & Phillips (1995b)	Davis (1935)
Diceroprocta delicata	Sanborn & Phillips (2001)	Sanborn & Phillips (2001)
Diceroprocta eugraphica	Sanborn & Phillips (1995b)	Drew et al. (1974)
Diceroprocta knighti	Sanborn & Phillips (1995b)	Davis (1917)
Diceroprocta olympi	Sanborn & Phillips (1995b)	Sanborn (pers. com.)
Diceroprocta semicincta	Sanborn & Phillips (1995b)	Davis (1925)
Diceroprocta swalei	Sanborn & Phillips (1995b)	Sanborn (pers. com.)
Diceroprocta texana	Sanborn & Phillips (1995b)	Sanborn (pers. com.)
Docidocercus chlorops	Morris et al (1988)	Morris et al. (1988)
Docidocercus gausodontus	Montealegre-Z & Morris (1999)	Montealegre-Z & Morris (1999)
Drosophila melanogaster	Bennet-Clark (1971)	Miller (2000)
Eilema japonica	Nakano et al. (2009)	Esaki et al. (1957)
Ephippiger epphipiger	Berg & Greenfield (2005)	Bellmann & Luquet (1993)
Ephippiger terrestris	Dumortier (1963)	Bellmann & Luquet (1993)
Euchaetes egle	Nakano et al. (2009)	Opler et al. (2009)
Grammia anna	Fullard & Fenton (1977)	Covell (2005)
Grammia arge	Fullard & Fenton (1977)	Covell (2005)
Grammia virgo	Fullard & Fenton (1977)	Covell (2005)
Gryllotalpa africana	De Graaf et al. (2005)	De Graaf et al. (2004)
Gryllotalpa australis	Kavanagh (1987)	Otte & Alexander (1983)
Gryllotalpa gryllotalpa	Bennet-Clark (1970)	Bennet-Clark (1970)
Gryllotalpa major	Hill (1998)	Hill (1998)
Gryllotalpa vineae	Bennet-Clark (1970)	Bennet-Clark (1970)
Gryllus bimaculatus	Simmons (1988)	Bellmann & Luquet (1993)
Gryllus campestris	Bennet-Clark (1970)	Bellmann & Luquet (1993)
Halysidota tessellaris	Fullard & Fenton (1977)	Covell (2005)
Haploa confusa	Fullard & Fenton (1977)	Covell (2005)
Haploa contigua	Fullard & Fenton (1977)	Covell (2005)
Herminia tarsicrinalis	Nakano et al. (2009)	Esaki et al. (1957)
Hypoprepia fucosa	Fullard & Fenton (1977)	Covell (2005)
Kawanaphila mirla	Mason & Bailey (1998)	Rentz (1993)
Kawanaphila nartee	Bailey & Simmons (1991)	Gwynne & Bailey (1988)
Kawanaphila yarraga	Mason & Bailey (1998)	Rentz (1993)
Leptophyes punctatissima	Zimmermann et al. (1989)	Bellmann & Luquet (1993)
Leurophyllum consanguineum	Morris et al (1988)	Morris et al. (1988)
Ligurotettix planum	Minckley et al. (1995)	Garcia (2006)
Lophaspis hebardi	Morris & Beier (1982)	Morris & Beier (1982)
Lycomorpha pholus	Fullard & Fenton (1977)	Covell (2005)
Lyristes chiricahua	Sanborn & Phillips (1995b)	Davis (1923)
Lyristes duryi	Sanborn & Phillips (1995b)	Davis (1917)
Lyristes inauditus	Sanborn & Phillips (1995b)	Drew et al. (1974)
Lyristes superbus	Sanborn & Phillips (1995b)	Drew et al. (1974)
Lyristes texanus	Sanborn & Phillips (1995b)	Sanborn (pers. com.)
Lyristes winemanna	Sanborn & Phillips (1995b)	Sanborn (pers. com.)
Mantis religiosa	Hill (2007)	Sueur (pers. observ.)
Mecopoda chirper	Nityananda & Balakrishnan (2008)	Nityananda & Balakrishnan (2006)

Mygalopsis marki	Römer & Bailey (1986)	WAISC (2010)
Neocicada chisos	Sanborn & Phillips (1995b)	Sanborn et al. (2005)
Neocicada hieroglyphica	Sanborn & Phillips (1995b)	Sanborn et al. (2005)
Neoconocephalus affinis	Brush et al. (1985)	Walker (2009)
Oecanthus quadripunctatus	Forrest (1991)	Walker (2009)
Okanagana hesperia	Sanborn & Phillips (1995b)	Drew et al. (1974)
Okanagana pallidula	Sanborn & Phillips (1995b)	Davis (1917)
Okanagana striatipes	Sanborn & Phillips (1995b)	Davis (1930)
Okanagana utahensis	Sanborn & Phillips (1995b)	Davis (1919)
Okanagodes gracilis	Sanborn & Phillips (1995b)	Davis (1919)
Okanagodes terlingua	Sanborn & Phillips (1995b)	Sanborn (pers. com.)
Ostrinia furnacalis	Nakano et al. (2009)	Esaki et al. (1957)
Oxypleura lenihani	Villet (1987)	Villet (1987)
Pacarina puella	Sanborn & Phillips (1995b)	Drew et al. (1974)
Palpita nigropunctalis	Nakano et al. (2009)	Esaki et al. (1957)
Panacanthus gibbosus	Montealegre-Z & Morris (2004)	Montealegre-Z & Morris (2004)
Panacanthus intensus	Montealegre-Z & Morris (2004)	Montealegre-Z & Morris (2004)
Panacanthus pallicornis	Montealegre-Z & Morris (2004)	Montealegre-Z & Morris (2004)
Panoploscelis specularis	Montealegre-Z et al. (2003)	Montealegre-Z et al. (2003)
Panoscelis specularis	Montealegre-Z et al. (2003)	Montealegre-Z et al. (2003)
Parascopioricus cordillericus	Montealegre-Z & Morris (1999)	Montealegre-Z & Morris (1999)
Phragmatobia fuliginosa	Fullard & Fenton (1977)	Covell (2005)
Platypleura argentata	Villet (1987)	Villet (1987)
Platypleura maytenophila	Villet (1987)	Villet (1987)
Platypleura zuluensis	Villet (1987)	Villet (1987)
Psorodonotus illyricus	Keuper et al. (1988)	Keuper et al. (1988)
Pycna semiclara	Villet (1987)	Villet (1987)
Pyrrharctia isabella	Fullard & Fenton (1977)	Covell (2005)
Rufocephalus sp.	Bailey et al. (2001)	Bailey et al. (2001)
Scapteriscus borelii	Forrest (1991)	Nickle (2003)
Scapteriscus vicinus	Forrest (1991)	Walker (2009)
Sciarasaga quadrata	Römer & Bailey (1998)	Römer & Bailey (1998)
Scopioricus spatulatus	Montealegre-Z & Morris (1999)	Montealegre-Z & Morris (1999)
Scopiorinus carinulatus	Morris & Beier (1982)	Morris & Beier (1982)
Scopiorinus impressopunctatus	Morris & Beier (1982)	Morris & Beier (1982)
Spilosoma punctarium	Nakano et al. (2009)	Esaki et al. (1957)
Spodoptera litura	Nakano et al. (2009)	Esaki et al. (1957)
Spoladea recurvalis	Nakano et al. (2009)	Esaki et al. (1957)
Symmoracma minoralis	Heller & Krahe (1994)	Heller & Krahe (1994)
Teleogryllus commodus	Kavanagh (1987)	Otte & Alexander (1983)
Teleutias fasciatus	Montealegre-Z & Morris (1999)	Montealegre-Z & Morris (1999)
Tettigonia cantans	Latimer & Schatral (1986)	Keuper et al. (1988)
Tettigonia viridissima	Keuper et al. (1988)	Keuper et al. (1988)
Tibicina corsica fairmairei	Sueur & Sanborn (2003)	Sueur (2002)
Tibicina garricola	Sueur & Sanborn (2003)	Sueur (2002)
Tibicina tomentosa	Sueur & Sanborn (2003)	Sueur (2002)
Trichotettix pilosula	Montealegre-Z & Morris (1999)	Montealegre-Z & Morris (1999)
Triencentrus atrosignatus	Montealegre-Z & Morris (1999)	Montealegre-Z & Morris (1999)
Tympanistalna gastrica	Michelsen & Fonseca (2000)	Fonseca & Bennet-Clark (1998)
Typophyllum bolivari	Morris et al (1988)	Morris et al. (1988)
Typophyllum mortuifolium	Morris et al (1988)	Morris et al. (1988)
Typophyllum zingara	Montealegre-Z & Morris (1999)	Montealegre-Z & Morris (1999)
Uchuca amacayaca	Montealegre-Z & Morris (2003)	Montealegre-Z & Morris (2003)

Mammal

Arctocephalus pusillus doriferus	Tripovitch et al. (2008)	Swolgaard (2002)
Arctocephalus tropicalis	Charrier et al. (2002)	Hiller (2000)
**Balaena mysticetus*	Kuperman & Roux (2006)	Justice (2002)
**Balaenoptera musculus*	Kuperman & Roux (2006)	Dewey & Fox (2002)
**Balaenoptera physalus*	Kuperman & Roux (2006)	Fox (2001)
Bison bison	Wyman et al. (2008)	Newell & Sorin. (2003)
Callithrix jacchus	Brumm et al. (2004)	Cover (2000)
Elephas maximus	Payne et al. (1986)	Ciszek (1999)
**Eschrichtius robustus*	Kuperman & Roux (2006)	Nowak (2003)
**Eubalaena australis*	Kuperman & Roux (2006)	Smith (2000)
**Hippopotamus amphibius*	Barklow (2002)	Shefferly (2001)
Homo sapiens	Ferguson et al (2008)	Australian Bureau of Statistics (1998)
Homo sapiens	Pinczower & Oates (2005)	Australian Bureau of Statistics (1998)
Homo sapiens	Pinczower & Oates (2005)	Australian Bureau of Statistics (1998)
Loxodonta africana	Poole et al. (1988)	Norwood (2002)
**Megaptera novaeangliae*	Thompson et al. (1986)	Spitz et al. (2002)
Mirounga angustirostris	Sanvito & Galimberti (2003)	Sanvito & Galimberti (2003)
Mirounga leonina	Sanvito & Galimberti (2003)	Sanvito & Galimberti (2003)
Myotis septentrionalis	Miller & Treat (1993)	Ollendorff (2002)
Neophoca cinerea	Charrier et al. (2009)	Hoglund (2003)
**Orcinus orca*	Miller (2006)	Burnett et al. (2009)
Papio cynocephalus ursinus	Kitchen et al. (2003)	Kitchen et al. (2003)
**Physeter macrocephalus*	Kuperman & Roux (2006)	Nowak (2003)
Saccopteryx bilineata	Behr et al. (2006)	Charles-Dominique et al. (2001)
**Stenella longirostris*	Kuperman & Roux (2006)	Bull (1999)
**Tursiops truncatus*	Au (1993)	Jenkins & Myers (2009)

Reptile

Alligator mississipiensis	Barklow (2004)	Pajerski et al. (2000)
Gekko gekko	Brillet & Paillette (1991)	Corl (1999)

References

Ahmed T (2003) *Pygoscelis papua*, Animal Diversity Web. Accessed September 02, 2010 at http://animaldiversity.ummz.umich.edu/site/accounts/information/Pygoscelis_papua.html

AmphibiaWeb (2010) Information on amphibian biology and conservation. Berkeley, California. Accessed September 02, 2010 at http://amphibiaweb.org/

Au WW L (1993) The Sonar of Dolphins. New York: Springer-Verlag.

Au WWL, Banks K (1998) The acoustics of the snapping shrimp *Synalpheus parneomeris* in Kaneohe Bay. Journal of the Acoustical Society of America 103: 41-47.

Aubin T, Jouventin P (1998) Cocktail-party effect in king penguin colonies. Proceedings of the Royal Society of London, B 265: 1665-1673.

Australian Bureau of Statistics (1998) How Australians measure up. Canberra: ABS, Commonwealth of Australia.

Bailey WJ, Simmons LW (1991) Male-male behavior and sexual dimorphism of the ear of a

Zaprochiline Tettigoniid (Orthoptera, Tettigoniidae). Journal of Insect Behavior 4: 51-64.

Bailey WJ, Bennet-Clark HC, Fletcher NH (2001) Acoustics of a small Australian burrowing cricket: the control of low-frequency pure-tone songs. Journal of Experimental Biology 204: 2827-2841.

Barklow WE (2004) Amphibious communication with sound in hippos, *Hippotamus amphibius*. Animal Behaviour 68: 1125-1132.

Behr O, von Helversen O, Heckel G, Nagy M, Voigt CC, Mayer F (2006) Territorial songs indicate male quality in the sac-winged bat *Saccopteryx bilineata* (Chiroptera, Emballonuridae). Behavioral Ecology 17: 810-817.

Bellmann H, Luquet G (1993) Guide des sauterelles, grillons et criquets d'Europe occidentale. Paris: Delachaux & Niestlé.

Bennet-Clark HC, Young Y (1998) Sound radiation by the bladder cicada *Cystosoma saundersii*. Journal of Experimental Biology 201: 701-715.

Bennet-Clark HC (1970) The mechanism and efficiency of sound production in mole crickets. Journal of Experimental Biology 52: 619-652.

Bennet-Clark HC (1971) Acoustics of insect song. Nature 234: 255-259.

Berg A, Greenfield MD (2005) Sexual selection in insect choruses: influences of call power and relative timing. Journal of Insect Behavior 18: 59-75.

Brackenbury JH (1979) Power capabilities of the avian sound-producing system. Journal of Experimental Biology 78: 163-166.

Brenowitz EA (1982) The active space of red-winged blackbird song. Journal of Comparative Physiology A 147: 511ï 522.

Briefer E, Aubin T, Lehongre K, Rybak F (2008) How to identify dear-enemies: the group signature in the complex song of the skylark *Alauda arvensis*. Journal of Experimental Biology 211: 317-326.

Brillet C, Paillette M (1991) Acoustic signals of the nocturnal lizard *Gekko gecko*; analysis of the 'long complex sequence'. Bioacoustics 3: 33-44.

Brown CH (1989) The measurement of vocal amplitude and vocal radiation pattern in blue monkeys and grey-cheeked mangabeys. Bioacoustics 1: 253-271

Brumm H, Voss K, Köllmer I, Todt D (2004) Acoustic communication in noise: regulation of call characteristics in a New World monkey. Journal of Experimental Biology 207: 443-448.

Brush JS, Gian VG, Greenfield MD (1985) Phonotaxis and aggression in the coneheaded katydid *Neoconocephalus affinis*. Physiological Entomology 10: 23-32.

Bull J (1999) *Stenella longirostris*. Animal Diversity Web. Accessed September 02, 2010 at http://animaldiversity.ummz.umich.edu/site/accounts/information/Stenella_longirostris.html

Burnett E, Francl K (2009) Orcinus orca. Animal Diversity Web. Accessed September 02, 2010 at

http://animaldiversity.ummz.umich.edu/site/accounts/information/Orcinus_orca.html

Charles-Dominique P, Brosset A., Jouard S (2001) Atlas des chauves-souris de Guyane. Paris: Editions MNHN.

Charrier, I, Sturdy, C (2005) Coding of the species-specific recognition: what makes a Blacked-capped chickadee chick-a-dee call? Behavioral Processes 70: 271-281.

Charrier I, Mathevon N, Jouventin P (2002) How does a fur seal mother recognize the voice of her pup? An experimental studi of *Arctocephalus tropicalis*. Journal of Experimental Biology 205: 603-612.

Charrier I, Mathevon N, Jouventin P, Aubin T (2001) Acoustic communication in a Black-Headed Gull colony: how do chicks identify their parents? Ethology 107: 961-974.

Charrier I, Pitcher BJ, Harcourt RG (2009) Vocal recognition of mothers by Australian sea lion pups: individual signature and environmental constraints. Animal Behaviour 78: 1127-1134.

Ciszek D (1999) *Elephas maximus*, Animal Diversity Web. Accessed September 02, 2010 at http://animaldiversity.ummz.umich.edu/site/accounts/information/Elephas_maximus.html

Combos V, Francl K (2008) *Pygoscelis adeliae*. Animal Diversity Web. Accessed September 02, 2010 at http://animaldiversity.ummz.umich.edu/site/accounts/information/Pygoscelis_adeliae.html

Conant R, Collins JT (1991) A field guide to Reptiles and Amphibians. Eastern and central north America. Boston: Peterson Field Guides.

Connaughton M, Taylor M, Fine M (2000) Effects of fish size and temperature on weakfish disturbance calls: implications for the mechanism of sound generation. Journal of Experimental Biology 203: 1503-1512.

Cook RD, Weisberg S (1982) Residuals and influence in regression. Chapman & Hall.

Corl, J. 1999 *Gekko gecko*. Animal Diversity Web. Accessed September 02, 2010 at http://animaldiversity.ummz.umich.edu/site/accounts/information/Gekko_gecko.html

Covel C (2005) A field guide to moths of Eastern North America. Martinsville: Virginia Museum of Natural History, Special Publication No. 12.

Cover S (2000) *Callithrix jacchus*. Animal Diversity Web. Accessed September 02, 2010 at http://animaldiversity.ummz.umich.edu/site/accounts/information/Callithrix_jacchus.html

Curé C, Aubin T, Mathevon T (2009) Acoustic convergence and divergence in two sympatric burrowing nocturnal seabirds. Biological Journal of the Linnean Society 96: 115-134.

Dambach M, Gras A (1995) Bioacoustics of a miniature cricket, *Cycloptiloides canariensis* (Orthoptera : Gryllidae : Mogoplistinae). Journal of Experimental Biology 198: 721-728.

Davis WT (1917) Sonoran cicadas collected by Harry H. Knight, Dr. Joseph Bequaert and others with descriptions of new species. Journal of the New York Entomological Society 25: 203-225.

Davis WT (1919 Cicadas of the genera *Okanagana*, *Tibicinoides* and *Okanagodes*, with descriptions

of several new species. Journal of the New York Entomological Society 27: 179-223.

Davis WT (1921 Records of cicadas from North America with descriptions of new species. Journal of the New York Entomological Society 19: 1-16.

Davis WT (1923 Notes on North American cicadas with descriptions of new species. Journal of the New York Entomological Society 31: 1-15.

Davis WT (1925) *Cicada tibicen*, a South American species with records and descriptions of North American cicadas. Journal of the New York Entomological Society 33 35-51.

Davis WT (1928) Cicadas belonging to the genus *Diceroprocta* with descriptions of new species. Journal of the New York Entomological Society 36: 439-458.

Davis WT (1930) The Distribution of cicadas in the United States with descriptions of new species. Journal of the New York Entomological Society 38: 53-72.

Davis WT (1935) New cicadas with notes on North American and West Indian species. Journal of the New York Entomological Society 43: 173-198.

De Graaf, J, Schoeman AS, Brandenburg RL (2005) Stridulation of *Gryllotalpa africana* (Orthoptera: Gryllotalpidae) on turf in South Africa. Florida Entomologist 88: 292-299.

Dewey T, Fox D (2002) *Balaenoptera musculus.* Animal Diversity Web. Accessed September 02, 2010 at http://animaldiversity.ummz.umich.edu/site/accounts/information/Balaenoptera_musculus.html

Drew WA, Spangler FL, Molnar D (1974) Oklahoma Cicadidae (Homoptera). Proceedings of Oklahoma Academy of Sciences 54: 90-97.

Dumortier B (1963) The physical characteristics of sound emissions in Arthropoda. In Acoustic behaviour in animals (ed. R.-G. Busnel R-G), pp. 346-373, Amsterdam: Elsevier.

Eades DC, Otte D, Cigliano MM, Braun H 2010 Orthoptera Species File Online. Version 2.0/3.5. Accessed September 02, 2010 at http://orthoptera.speciesfile.org.

Esaki T, Isshiki S, Inoue H, Mutuura A, Ogata M, Okagaki H (1957) *Icones Heterocerorum Japonicorum in Coloribus Naturalibus*. Tokyo: Hoikusha.

Ferguson S, Kenny DT, Cabrera D (2008) Effects of training on time-varying spectral energy and sound pressure level in nine male classical singers. Journal of Voice 24: 39-46.

Fonseca PJ, Bennet-Clark HC (1998) Asymmetry of tymbal action and structure in a cicada: a possible role in the production of complex songs. Journal of Experimental Biology 201: 717-730.

Forrest TG (1991) Power output and efficiency of sound production by crickets. Behavioral Ecology 2: 327-338.

Forrest TG, Miller GL, Zagar JR (1993) Sound propagation in shallow water: implications for acoustic communication by aquatic animals. Bioacoustics 4: 259-270

Fox D (2001) *Balaenoptera physalus*. Animal Diversity Web. Accessed September 02, 2010 at

http://animaldiversity.ummz.umich.edu/site/accounts/information/Balaenoptera_physalus.html

Fox J (2002) An R and S-PLUS companion to applied regression. Sage: Thousand Oaks.

Fullard JH, Fenton MB (1977) Acoustic and behavioural analyses of the sounds produced by some species of Nearctic Arctiidae (Lepidoptera). Canadian Journal of Zoology 55: 1213-1224.

Fullard JH (1977) Variability of sexual dimorphism in the sounds of *Cycnia tenera* Hübner (Lepidoptera: Arctiidae). Journal of the New York Entomological *Society* 85: 21-25.

Garcia ER (2006) An annotated checklist of some orthopteroid insects of Mapimi biosphere reserve (Chihuahuan desert), Mexico. Acta Zoológica Mexicana (nueva serie) 22: 131-149.

Gerhardt HC (1975) Sound pressure levels and radiation patterns of the vocalizations of some north american frogs and toads. Journal of Comparative Physiology A 102: 1-12.

Gwynne DT, Bailey WJ (1988) Mating System, Mate Choice and Ultrasonic Calling in a Zaprochiline Katydid (Orthoptera: Tettigoniidae). Behaviour 105: 202-223.

Heller KG, Achmann R (1993) The ultrasonic song of the moth *Amyna natalis* (Lepidoptera: Noctuidae: Acontiinae). Bioacoustics 5: 89-97.

Heller KG, Krahe R (1994) Sound production and hearing in the pyralid moth Symmoracma minoralis. Journal of Experimental Biology 187: 101-111.

Hill PSM (1998) Environmental and social influences on calling effort in the prairie mole cricket (*Gryllotalpa major*). Behavioral Ecology 9: 101-108.

Hill SA (2007) Sound generation in *Mantis religiosa* (Mantodea: Mantidae): stridulatory structures and acoustic signal. Journal of Orthoptera Research 16: 35-49.

Hiller C (2000) *Arctocephalus tropicalis*. Animal Diversity Web. Accessed September 02, 2010 at http://animaldiversity.ummz.umich.edu/site/accounts/information/Arctocephalus_tropicalis.html

Hoglund K (2003) *Neophoca cinerea*. Animal Diversity Web. Accessed September 02, 2010 at http://animaldiversity.ummz.umich.edu/site/accounts/information/Neophoca_cinerea.html

Jenkins J, Myers P (2009) *Tursiops truncatus*. Animal Diversity Web. September 02, 2010 at http://animaldiversity.ummz.umich.edu/site/accounts/information/Tursiops_truncatus.html

Jonsson L (1994) Les oiseaux d'Europe. Paris: Nathan.

Jouventin P, Aubin T (2002) Acoustic systems are adapted to breeding ecologies: individual recognition in nesting penguins. Animal Behaviour 64: 747-757.

Jouventin P, Aubin T (2000) Acoustic convergence in the calls of two nocturnal burrowing seabirds. Experiments with a penguin (*Eudyptula minor*) and a shearwater (*Puffinus tenuirostris*). Ibis 142: 645-656.

Justice J (2002) *Balaena mysticetus*. Animal Diversity Web. September 02, 2010 at http://animaldiversity.ummz.umich.edu/site/accounts/information/Balaena_mysticetus.html

Kavanagh MW (1987) The efficiency of sound production in two cricket species, *Gryllotalpa australis* and *Teleogryllus commodus* (Orthoptera, Grylloidea). Journal of Experimental Biology 130: 107-119.

Keuper A, Weidemann S, Kalmring K, Kaminski D (1988) Sound production and sound emission in seven species of European tettigoniids: Part 1. The different parameters of the song: their relation to the morphology of the bushcricket. Bioacoustics 1: 31-48.

Kitchen DM, Seyfath RM, Fischer J, Cheney J (2003) Loud calls as indicators of dominance in male baboons (*Papio cynocephalus ursinus*). Behavioral Ecology and Sociobiology 53: 374-384.

Korc M, Fraser A (2006) *Aptenodytes patagonicus*. Animal Diversity Web. Accessed September 02, 2010 at http://animaldiversity.ummz.umich.edu/site/accounts/information/Aptenodytes_patagonicus.html

Kuperman WA, Roux P (2006) Underwater acoustics. In Handbook of Acoustics (ed. T. D. Rossing), pp. 149-204, Paris: New York.

Lambiris AJL (1989) The frogs of Zimbabwe. Turin: Museo Regionale di Scienze naturali.

Latimer W, Schatral A (1986) Information cues used in male competition by *Tettigonia cantans* (Orthoptera: Tettigoniidae). Animal Behaviour 34: 162-168.

Lengagne T, Slater PJB (2002) The effects of rain on acoustic communication : tawny owls have good reason for calling less in wet weather. Proceedings of the Royal Society of London, B 269: 2121-2125.

Lindsey TR (1986) The seabirds of Australia. Sydney: Angus and Robertson, and the National Photographic Index of Australian Wildlife.

MacNally R, Young D (1981) Song energetics of the bladder cicada, *Cystosoma saundersii*. Journal of Experimental Biology 90: 185-196.

Marquez R, Moreira C, do Amaral JPS, Pargana JM, Crespo EG (2005) Sound pressure level of advertisement calls of *Hyla meridionalis* and *Hyla arborea*. Amphibia-Reptilia 26: 391-395.

Maruska KP, Boyle KS, Dewan LR, Tricas TC (2007) Sound production and spectral hearing sensitivity in the Hawaiian sergeant damselfish, *Abudefduf abdominalis*. Journal of Experimental Biology 210: 3990-4004.

Mason AC, Bailey WJ (1998) Ultrasound hearing and male-male communication in Australian katydids (Tettigoniidae: Zaprochilinae). Physiological Entomology 23: 139-149.

Michelsen A, Fonseca P (2000) Spherical sound radiation patterns of singing grass cicadas, *Tympanistalna gastrica*. Journal of comparative Physiology A, 186: 163-168.

Miller C (2000) *Drosophila melanogaster*. Animal Diversity Web. Accessed September 02, 2010 at http://animaldiversity.ummz.umich.edu/site/accounts/information/Drosophila_melanogaster.html

Miller LA, Treat AE (1993) Field recordings of echolocation and social signals from the gleaning bat *Myotis septentrionalis*. Bioacoustics 5: 67-87.

Miller PJO (2006) Diversity in sound pressure levels and estimated active space of resident killer whale vocalizations. Journal of Comparative Physiology A 192: 449ï 459.

Minckley RL, Greenfield MD, Tourtellot MK (1995) Chorus structure in tarbush grasshoppers: inhibition, selective phonoresponse and signal competition. Animal Behaviour 50: 579-594.

Mohl B, Wahlberg M, Madsen PT, Heerfordt A, Lund A. (2003) The monopulsed nature of Sperm Whale clicks. Journal of the Acoustical Society of America 114: 1143ï 1154.

Montealegre-Z F, Morris GK (1999) Songs and systematics of some Tettigoniidae from Colombia and Ecuador I. Pseudophyllinae (Orthoptera). Journal of Orthoptera Research 8: 163-236.

Montealegre-Z F, Morris GK (2003) Uchuca Giglio-Tos, *Dectinomina* Caudell and their allies (Orthoptera: Tettigoniidae: Conocephalinae). Transactions of the American Entomological Society 3-4: 503-537.

Montealegre-Z F, Morris GK (2004) The spiny devil katydids, *Panacanthus* Walker (Orthoptera: Tetigoniidae): an evolutionary study of acoustic behaviour and morphological traits. Systematic Entomology 29: 21-57.

Montealegre-Z F, Guerra PA, Morris GK (2003) *Panoploscelis specularis* (Orthoptera: Tettigoniidae: Pseudophyllinae): extraordinary female sound generator, male description, male protest and calling signals. Journal of Orthoptera Research 12: 173-181.

Morris GK, Beier M (1982) Song structure and description of some Costa Rican katydids (Orthoptera: Tettigoniidae). Transactions of the American Entomological Society 108: 287-314.

Morris GK, Klimas DE, Nickle DA (1988) Acoustic signals and systematics of False-Leaf Katydids from Ecuador (Orthoptera, Tettigoniidae, Pseudophyllinae). Transactions of the American Entomological Society 114: 215-263.

Morris GK, Mason AC, Wall MP (1994) High ultrasonic and tremulation signals in neotropical katydids (Orthoptera: Tettigoniidae). Journal of Zoology, London 233: 129-163.

Murphy MD, Taylor RG (1989) Reproduction and growth of black drum, *Pogonias cromis*, in Northeast Florida. Northeast Gulf Sciences,10: 127-137.

Nakano R, Takanashi T, Fujii T, Skals N, Surlykke A, Ishikawa Y (2009) Moths are not silent, but whisper ultrasonic courtship songs. Journal of Experimental Biology 212: 4072-4078.

Narins PM, Hurley DD (1982) The relationship between call intensity and function in the Puerto Rican coqui. *Herpetologica* 38: 287-295.

Newell T, Sorin A (2003) *Bison bison*. Animal Diversity Web. Accessed September 02, 2010 at at http://animaldiversity.ummz.umich.edu/site/accounts/information/Bison_bison.html

Nickle DA (2003) A Revision of the mole cricket genus *Scapteriscus* with the description of a morphologically similar new genus (Orthoptera: Gryllotalpidae: Scapteriscinae). Transactions of the American Entomological Society 129: 411-485.

Nityananda V, Balakrishnan R (2006) A diversity of songs among morphologically indistinguishable katydids of the genus *Mecopoda* (Orthoptera: Tettigoniidae) from Southern India. Bioacoustics 15:

223-250.

Nityananda V, Balakrishnan R (2008) Leaders and followers in katydid choruses in the field: call intensity, spacing and consistency. Animal Behaviour 76: 723-735.

Norwood L (2002) *Loxodonta africana*. Animal Diversity Web. Accessed September 02, 2010 at http://animaldiversity.ummz.umich.edu/site/accounts/information/Loxodonta_africana.html

Nowak RM (2003) Walker's marine Mammals of the world. Johns Hopkins University.

Ollendorff J (2002) *Myotis septentrionalis*. Animal Diversity Web. Accessed September 02, 2010 at http://animaldiversity.ummz.umich.edu/site/accounts/information/Myotis_septentrionalis.html

Opler PA, Lotts K, Naberhaus T (2009) Butterflies and Moths of North America. Bozeman, MT: Big Sky Institute. Accessed September 02, 2010 at http://www.butterfliesandmoths.org/

Otte D, Alexander RD (1983) *The Australian crickets (Orthoptera: Gryllidae)*. Pennsylvania: Academy of Natural Sciences of Philadelphia, Allen Press.

Pajerski L, Schechter B, Street R (2000) *Alligator mississippiensis*. Animal Diversity Web. Accessed September 02, 2010 at http://animaldiversity.ummz.umich.edu/site/accounts/information/Alligator_mississippiensis.html

Passmore NI (1981) Sound levels of mating calls of some African frogs. Herpetologica 37: 166-171.

Patek SN, Shipp LE, Staaterman ER (2009) The acoustics and acoustic behavior of the California spiny lobster (*Panulirus interruptus*). Journal of the Acoustical Society of America 125: 3434-3443.

Payne KB, Langbauer Jr WR, Thomas EM (1986) Infrasonic calls of the Asian elephant (*Elephas maximus*). Behavioral Ecology and Sociobiology 18: 297-301.

Penna M, Solis MA (2005) Frog call intensities and sound propagation in the South American temperate forest region. Behavioral Ecology and Sociobiology 43: 371-381.

Peterson RT (1980) Eastern birds. Boston: Peterson Field Guides.

Pinczower R, Oates J (2005) Vocal projection in actors: the long-term average spectral features that distinguish comfortable acting voice from voicing with maximal projection in male actors. Journal of *Voice* 19: 440-453.

Poole JH, Payne KB, Langbauer Jr WR, Moss CM (1988) The social contexts of some very low frequency calls of African elephants. Behavioral Ecology and Sociobiology 22: 385-392.

Quick H (2001) *Eudyptula minor*. Animal Diversity Web. Accessed September 02, 2010 at http://animaldiversity.ummz.umich.edu/site/accounts/information/Eudyptula_minor.html

R Development Core Team (2010) R: A Language and Environment for Statistical Computing. Vienna, Austria, Accessed September 02, 2010 at http://www.R-project.org

Rentz DC (1993) Tettigoniidae of Australia. Volume 2. The Austrosaginae, Zaprochilinae and Phasmodinae. Melbourne: CSIRO.

Reynolds K (2001) *Eudyptes chrysolophus*. Animal Diversity Web. Accessed September 02, 2010 at http://animaldiversity.ummz.umich.edu/site/accounts/information/Eudyptes_chrysolophus.html

Römer H, Bailey W (1986) Insect hearing in the field. II. Male spacing behaviour and correlated acoustic cues in the bushcricket *Mygalopsis marki*. Journal of comparative Physiology A 159: 627-638.

Römer H, Bailey W (1998) Strategies for hearing in noise: peripheral control over auditory sensitivity in the bushcricket *Sciarasaga quadrata* (Austrosaginae: Tettigoniidae). Journal of Experimental Biology 201: 1023-1033.

Ryan MJ (1985) Energetic efficiency of vocalization by the frog Physalaemus pustulosus. Journal of Experimental Biology 116: 47-52.

Sanborn AF, Heath MS, Sueur J, Phillips PK (2005) The genus *Neocicada* Kato, 1932 (Hemiptera: Cicadomorpha: Cicadidae), with descriptions of three new species. Systematic Entomology 30: 191-207.

Sanborn AF, Phillips PK (1995) Scaling of sound pressure level and body size in cicadas (Homoptera: Cicadidae; Tibicinidae). Annals of the Entomological Soceity of America 88: 479-484.

Sanborn AF, Phillips PK (2001) Re-evaluation of the *Diceroprocta delicata* (Homoptera : Cicadidae) species complex. Annals of the Entomological Society of America 94: 159-165.

Sanborn AF, Heath JE, Heath MS (2009) Long-range sound distribution and the calling song of *Beameria venosa* (Uhler) (Hemiptera: Cicadidae). Southwestern Naturalist 54: 24-30.

Sanvito S, Galimberti F (2003) Source level of male vocalisations in the genus *Mirounga*: repeatability and correlates. Bioacoustics 14: 47-59.

Searby A, Jouventin P, Aubin T (2004) Acoustic recognition in macaroni penguins : an original signature system. Animal Behaviour 67: 615-625.

Sebe F, Dubosq J, Ligout S, Aubin T, Poindron P (2010) Early vocal recognition of mother by lambs: contribution of low- and high-frequency vocalizations. Animal Behaviour 79: 1055-1066.

Shefferly N (2001) *Hippopotamus amphibius*. Animal Diversity Web. Accessed September 02, 2010 at http://animaldiversity.ummz.umich.edu/site/accounts/information/Hippopotamus_amphibius.html

Simmons LW (1988) The calling song of the field cricket, *Gryllus bimaculatus* (De Geer) : constraints on transmission and its role in intermale competition and female choice. Animal Behaviour 36: 380-394.

Smith J (2000) *Eubalaena australis*. Animal Diversity Web. Accessed September 02, 2010 at http://animaldiversity.ummz.umich.edu/site/accounts/information/Eubalaena_australis.html

Spitz SS, Herman LM, Pack AA, Deakos MH (2002) The relation of body size of male humpback whales to their social roles on the Hawaiian winter grounds. Canadian Journal of Zoology 80: 1938ï 1947

Staaden van MJ, Römer H (1997) Sexual signaling in Bladder grasshoppers: tactical design for maximizing calling range. Journal of Experimental Biology 200: 2597-2608.

Sueur J (2002) Eco-éthologie de la communication sonore des cigales: le modèle *Tibicina* Amyot, 1847 (Hemiptera, Cicadidae, Tibicininae). PhD Thesis, Ecole Pratique des Hautes Etudes, Paris.

Sueur J, Aubin T, Simonis C (2008) Seewave: a free modular tool for sound analysis and synthesis. Bioacoustics 18: 213-226.

Sueur J, Sanborn AF (2003) Ambient temperature and sound power of cicada calling sound (Hemiptera Cicadidae *Tibicina*). Physiological Entomology 28: 340-343.

Swolgaard C (2002) *Arctocephalus australis*, Animal Diversity Web. Accessed September 02, 2010 at http://animaldiversity.ummz.umich.edu/site/accounts/information/Arctocephalus_australis.html

Thompson PO, Cummings WC, Ha SJ (1986) Sounds, source levels, and associated behavior of humpback whales, southeast Alaska. *Journal of the Acoustical Society of America*, 80, 735-740.

Tripovich JS, Charrier I, Rogers TL, Canfield R, Arnould JPY (2008) Who goes there? Differential responses to neighbor and stranger vocalizations in male Australian fur seals. Marine Mammal Science 24: 913-928.

Villet M (1987) Sound pressure levels of some African cicadas (Homoptera : Cicadoidea). Journal of the Entomological Society of South Africa 50: 269-273.

Walker TJ (1973) Systematics and acoustic behavior of U.S. and Caribbean short-tailed crickets (Orthoptera: Gryllidae: Anurogryllus). Annals of the Entomological Society of America 66: 1269-1277.

Walker TJ (2009) Singing Insects of North America. Accessed September 02, 2010 at http://www.entnemdept.ufl.edu/walker/buzz/index.html

Waser PM, Waser MS (1977) Experimental studies of primate vocalization - specializations for long-distance propagation. Zeitschrift für Tierpshychologie 43: 239-263.

WAISC (2010) Western Australian Insect Study Society. http://www.museum.wa.gov.au/waiss/

Wilczynski W, Rand AS, Ryan MJ (2001) Evolution of calls and auditory tuning in the *Physalaemus pustulosus* species group. Brain, Behaviour and Evolution 58: 137-151.

Wright AH, Wright AA (1949) Handbook of frogs and toads of the United States and Canada. New York: Comstock Publishing Associates.

Wyman MT, Mooring MS, McCowan B, Penedo MCT, Hart LA (2008) Amplitude of bison bellows reflects male quality, physical condition and motivation. Animal Behaviour 76: 1625ï 1639.

Yager D (1992) Underwater acoustic communication in the African Pipid frog *Xenopus borealis*.. Bioacoustics 4: 1-24.

Young D (1990) Do cicadas radiate sound through their ear-drums? Journal of Experimental Biology 151: 41-56.

Zimmermann U, Rheinlaender J, Robinson D (1989) Cues for male phonotaxis in the duetting bushcricket *Leptophyes punctatissima*. Journal of comparative Physiology A 164: 621-628.
